# Analysis of the indications for and results of breast cancer screening by magnetic resonance imaging at a cancer center in Brazil

**DOI:** 10.1590/0100-3984.2023.0111-en

**Published:** 2024-04-06

**Authors:** Karina Kuhl Zoghbi, Vinicius Cardona Felipe, Luciana Graziano, Camila Souza Guatelli, Juliana Alves de Souza, Almir Galvão Vieira Bitencourt

**Affiliations:** 1 Graduate Program, A.C.Camargo Cancer Center, São Paulo, SP, Brazil; 2 Hospital Saúde da Mulher, Belém, PA, Brazil; 3 Department of Imaging, A.C.Camargo Cancer Center, São Paulo, SP, Brazil

**Keywords:** Mass screening, Breast neoplasms/diagnostic imaging, Magnetic resonance imaging/methods, Early detection of cancer/methods, Mammography, Programas de rastreamento, Neoplasias da mama/diagnóstico por imagem, Ressonância magnética/métodos, Detecção precoce do câncer/métodos, Mamografia

## Abstract

**Objective:**

To evaluate the indications for and results of magnetic resonance imaging (MRI) examinations for breast cancer screening at a cancer center in Brazil.

**Materials and Methods:**

This was a retrospective observational study, based on electronic medical records, of patients undergoing MRI for breast cancer screening at a cancer center in Brazil.

**Results:**

We included 597 patients between 19 and 82 years of age. The main indications for MRI screening were a personal history of breast cancer, in 354 patients (59.3%), a family history of breast cancer, in 102 (17.1%), and a confirmed genetic mutation, in 67 (11.2%). The MRI result was classified, in accordance with the categories defined in the Breast Imaging Reporting and Data System, as benign (category 1 or 2), in 425 patients (71.2%), probably benign (category 3), in 143 (24.0%), or suspicious (category 4 or 5), in 29 (4.9%). On MRI, 11 malignant tumors were identified, all of which were invasive carcinomas. Among those 11 carcinomas, six (54.5%) were categorized as minimal cancers (< 1 cm), and the axillary lymph nodes were negative in 10 (90.9%). The cancer detection rate was 18.4/1,000 examinations, and the positive predictive value for suspicious lesions submitted to biopsy was 37.9%.

**Conclusion:**

In our sample, the main indication for breast MRI screening was a personal history of breast cancer. The results indicate that MRI is a highly accurate method for the early detection of breast neoplasms in this population.

## Abstract (pt)

**Objetivo:**

Avaliar as indicações e resultados de exames de ressonância magnética (RM) para rastreamento de câncer de mama em um centro oncológico no Brasil.

**Materiais e Métodos:**

Estudo observacional, realizado mediante análise retrospectiva de pacientes submetidos a RM das mamas para rastreamento de câncer de mama, por meio de revisão do prontuário eletrônico em um centro oncológico.

**Resultados:**

Foram incluídas 597 pacientes com idade variando de 19 a 82 anos. As principais indicações para rastreamento foram história pessoal de câncer de mama em 354 (59,3%) pacientes, história familiar em 102 (17,1%) e mutação genética confirmada em 67 (11,2%). O resultado da RM foi benigno (BI-RADS 1 ou 2) em 425 (71,2%) pacientes, provavelmente benigno (BI-RADS 3) em 143 (24,0%) e suspeito (BI-RADS 4 ou 5) em 29 (4,9%). Foram identificados 11 tumores malignos na RM, todos carcinomas invasivos, porcentagem de cânceres “mínimos” (< 1 cm) de 54,5% e porcentagem de axila negativa de 90,9%. A taxa de detecção de câncer na RM foi 18,4/1000 exames e o valor preditivo positivo para as lesões suspeitas submetidas a biópsia foi 37,9%.

**Conclusão:**

A principal indicação para RM de rastreamento na nossa população foi história pessoal de câncer de mama. Os resultados mostraram que a RM constitui um método com alta acurácia para detecção precoce de neoplasias da mama nessa população.

## INTRODUCTION

Among all types of cancer, breast cancer has the highest mortality rate and is the cancer type that most affects women in Brazil^(1)^. Mammography is the method of choice for population-based breast cancer screening, with annual mammography being recommended for women between 40 and 75 years of age^(2)^. However, mammography has reduced sensitivity in some situations, especially in patients who have dense breasts or are at high risk for developing breast cancer, making it often necessary to employ other, complementary imaging methods for screening in this population^(3,4,5)^.

Magnetic resonance imaging (MRI) is the most sensitive method for diagnosing breast cancer. In addition, tumors detected by MRI have a more aggressive biological profile than do those diagnosed by mammography^(6)^. Since 2007, this method has been indicated for screening women who are at a high lifetime risk of developing breast cancer^(7)^. The main indications for screening with breast MRI are as follows^(8,9)^: having the BRCA1 or BRCA2 gene mutation, or having a first-degree relative proven to be a carrier of such a mutation; having a lifetime risk of ≥ 20%, as calculated with one of the mathematical models based on personal and family history; undergoing thoracic radiotherapy between 10 and 30 years of age; having a genetic mutation that increases the risk of breast cancer or having a first-degree relative with such a mutation; having a personal history of breast cancer or high-risk lesions; and having dense breasts^(10)^.

Although there are a number of international reference works, there are few data on MRI screening for breast cancer in Brazil. Therefore, the objective of this study was to evaluate the indications for and results of MRI for breast cancer screening at a referral center for cancer in Brazil.

## MATERIALS AND METHODS

This was a single-center, retrospective observational study of women undergoing MRI for breast cancer screening between January and December of 2020. The study was approved by the local research ethics committee (Reference no. 42654121.2.0000.5432). Because of the retrospective nature of the study, the requirement for informed consent was waived. For auditing purposes, breast MRI scans performed at our facility are classified as screening examinations or diagnostic examinations; only those indicated for screening were included in this study. Patients in whom the physical examination revealed abnormalities were excluded, as were those who had previously undergone conventional imaging tests, those for whom clinical data were unavailable, and those who had not been followed at the same facility.

Patients were selected through analysis of the data and images included in the electronic medical records and picture archiving system of the facility. Data from the electronic medical records were evaluated in order to identify risk factors for breast cancer in patients undergoing MRI screening and post-examination follow-up. For patients in whom a malignant tumor was identified during screening, histological and immunohistochemical data were considered, including those from percutaneous biopsy specimens and those from surgical specimens.

For patients without a personal history of breast cancer, risk was calculated by using the Tyrer-Cuzick model, version 8.0^(11)^. This model takes into account nonhereditary risk factors (e.g., age, weight, menstrual history, and reproductive history), family history, the presence of *BRCA* mutations, previous biopsy results, and breast density but does not consider a personal history of breast cancer. As previously described^(12)^, the risk of breast cancer was classified as low (< 15%), intermediate (15–20%) or high (> 20%).

The MRI scans were acquired in a 1.5-T scanner, before and after the use of intravenous contrast, using a conventional protocol that consisted of the following: an axial T1-weighted gradient-echo sequence, without fat saturation; sagittal short-tau inversion recovery sequences, with fat saturation, of both breasts; an axial diffusion-weighted echo-planar sequence using the array spatial sensitivity encoding technique, with b values of 0 s/mm^2^ and 750 s/mm^2^; and a dynamic study, consisting of five axial T1-weighted three-dimensional (3D) gradient-echo sequences with fat suppression, one performed before and four performed after the administration of paramagnetic contrast (gadolinium) at a dose of 0.1 mmol/kg of body weight, as well as sagittal T1-weighted 3D gradient-echo sequences, with fat saturation and high spatial resolution, of both breasts.

The data obtained were stored in a database for statistical analysis with the IBM SPSS statistics software package, version 20.0 (IBM Corp., Armonk, NY, USA). To compare qualitative variables, we used Pearson’s chisquare test with Yates’ correction or Fisher’s exact test, as appropriate. Values of *p* ≤ 0.05 were considered statistically significant. To evaluate the screening results, the population was divided into two groups: individuals with a personal history of breast cancer (surveillance) and those without (high-risk screening). As recommended in the American College of Radiology (ACR) BI-RADS lexicon^(13)^, we calculated the following variables:

– Abnormal interpretation rate (%): number of examinations/total examinations.
– Recall rate (%): number of abnormal examinations/total examinations.
– Cancer detection rate: number of breast carcinomas/ 1,000 examinations—expected result, 20–30.
– Positive predictive value 1 (PPV1) – abnormal screening examinations (%): number of breast carcinomas/number of examinations.
– Positive predictive value 2 (PPV2) – biopsy recommended (%): number of breast carcinomas/number of examinations indicating the need for biopsy—expected result, 15%.
– Positive predictive value 3 (PPV3) – biopsy performed (%): number of breast carcinomas/number of biopsies— expected result, 20–50%.
– Proportion of minimal cancers (%): number of carcinomas measuring < 1 cm or categorized as ductal carcinoma *in situ* (DCIS)/number of breast carcinomas identified in the study population—expected result, > 50%.
– Proportion of invasive carcinomas with node-negative axillae (%): number of invasive breast carcinomas without axillary metastasis/number of invasive breast carcinomas identified in the study population—expected result, > 80%.

## RESULTS

During the study period, 2,227 breast MRI examinations were performed at the facility. Of those, 624 (28.0%) were screening examinations and were initially selected. A total of 27 examinations were excluded: some because of incomplete data; some because they were duplicate records; and some because they were in patients with biopsy-proven malignancy (BI-RADS 6). Therefore, the final sample comprised 597 breast MRI examinations. The mean age of the patients was 48.8 ± 11.1 years (range, 19–82 years).

### Indications for MRI breast cancer screening

In our sample, the main indications for MRI were a personal history of breast cancer, in 354 patients (59.3%); a family history of breast cancer, in 102 (17.1%); a known mutation, in 67 (11.2%); dense breasts, in 14 (2.3%); and a history of radiotherapy, in 4 (0.7%). In 56 patients (9.4%), the indication for the test was not specified. Among the patients who underwent screening MRI for a known mutation, the main mutations observed were as follows: BRCA1 (n = 17); BRCA2 (n = 16); *P53* (n = 25); CHEK2 (n = 4); and other mutations (n = 5), including PALB2, CDH1, MLH1 (associated with Lynch syndrome), RET*,* and a variant of uncertain significance in the POLE gene.

Using the Tyrer-Cuzick model to assess the risk of breast cancer in the 243 patients without a personal history of breast cancer, we observed that 113 (46.5%) presented normal risk (< 15%), 40 (16.5%) presented intermediate risk (15–20%), and 90 (37.0%) presented high risk (> 20%). Table 1 shows the risk of developing breast cancer according to the indication for screening.

**Table 1:** Stratification of the risk for developing breast cancer, as estimated with the Tyrer-Cuzick model, according to the indication for screening MRI.

Indication for MRI	Tyrer-Cuzick risk of breast cancer			Total
	< 15% n (%)	15–20% n (%)	> 20% n (%)	
Family history	33 (32.4)	24 (23.5)	45 (44.1)	102
Known mutation	21 (31.3)	11 (16.4)	35 (52.2)	67
Dense breasts	9 (64.3)	2 (14.3)	3 (21.4)	14
Previous radiotherapy	3 (75.0)	0 (0.0)	1 (25.0)	4
Unspecified high risk	47 (83.9)	3 (5.3)	6 (10.7)	56
Total	113 (46.5)	40 (16.5)	90 (37.0)	243

### Results of MRI breast cancer screening

Among the 597 screening examinations evaluated, the results were classified, by category, as BI-RADS 1 in three cases (0.5%), BI-RADS 2 in 422 (70.7%), BI-RADS 3 in 143 (24.0%), BI-RADS 4 in 25 (4.2%), and BI-RADS 5 in four (0.7%). The abnormal interpretation rate was 28.8% (n = 172), and the recall rate was 4.9% (n = 29). The results of the breast MRI examinations are summarized, by type of screening, in Table 2. The abnormal interpretation rate was significantly lower among the examinations performed in women with a personal history of breast cancer than among those performed in women with no such history (20.9% vs. 40.3%; *p* < 0.001). However, there was no statistical difference between those two groups in terms of the recall rate (3.7% vs. 6.6%; *p* = 0.104).

**Table 2:** Breast MRI results according to the indication for the examination (surveillance vs. high-risk screening).

Indication	BI-RADS category			Total
	1 or 2 n (%)	3 n (%)	4 or 5 n (%)	
Surveillance	280 (79.1)	51 (17.2)	13 (3.7)	354
High-risk screening	145 (59.7)	82 (33.7)	16 (6.6)	243
Total	425 (71.2)	143 (24.0)	29 (4.9)	597

**Figure 1:**
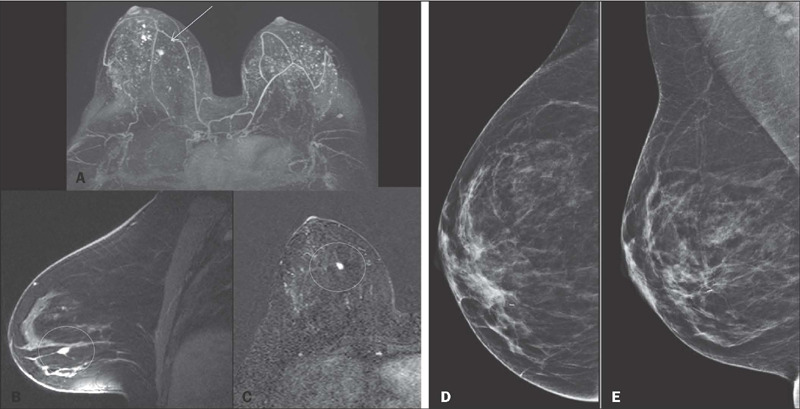
A 42-year-old patient who underwent mammography and MRI for breast cancer screening. MRI (**A–C**) showing an 8-mm mass in the inferomedial quadrant of the right breast (arrow and circles) that had not been identified on mammography (**D,E**) and was confirmed to be invasive breast carcinoma after biopsy.

Table 3 shows the main breast MRI findings described for the BI-RADS categories 3, 4 and 5. Table 4 shows how the MRI findings were subsequently investigated and the results of that investigation, by BI-RADS category. In our sample, 11 malignant tumors were identified on breast MRI (Table 5). One of those tumors is exemplified in Figure 1. Therefore, the cancer detection rate was 18.4/1,000 examinations—16.9/1,000 examinations among the patients with a personal history of breast cancer and 20.5/1,000 examinations among the patients with no such history. In the sample as a whole, the PPV1 was 6.4%, given that malignancy was identified in 11 of the 172 screening examinations with abnormal findings, whereas it was 8.1% and 5.1% for the examinations performed in women with and without a personal history of breast cancer, respectively (malignancy being identified in six of the 74 and in five of the 98, respectively). Among the 29 patients who underwent biopsy, the PPV2 and PPV3 were both 37.9%, both being 46.2% among the 13 patients with a personal history of breast cancer and 31.3% among the 16 patients without. Among the 11 malignant tumors identified, six (54.5%) were classified as minimal cancers (< 1 cm) and 10 (90.9%) were in patients with node-negative axillae.

**Table 3:** Main abnormal findings on breast MRI, by BI-RADS category.

BI-RADS category	Mass n (%)	Non-mass enhancement n (%)	Suspicious lymph node n (%)	Other n (%)	Total n (%)
3	54 (37.8)	86 (60.1)	2 (1.4)	1 (0.7)	143
4	12 (48.0)	11 (44.0)	1 (4.0)	1 (4.0)	25
5	4 (100)	0 (0.0)	0 (0.0)	0 (0.0)	4
Total	70 (40.7)	97 (56.4)	3 (1.7)	2 (1.2)	172

**Table 4:** Type of investigation of the MRI findings, by BI-RADS category.

BI-RADS category	Type of investigation			Malignancy n (%)	Total
	None n (%)	Ancillary tests n (%)	Biopsy n (%)		
3	45 (31.7)	84 (59.2)	13 (9.2)	0 (0.0%)	142
4	0 (0.0)	3 (12.0)	22 (88.0)	8 (32.0%)	25
5	0 (0.0)	0 (0.0)	4 (100)	3 (75.0%)	4
Total	45 (26.3)	87 (50.9)	39 (22.8)	11 (6.4%)	171

**Table 5:** Characteristics of patients in whom malignant tumors were detected on breast MRI.

Age (years)	Indication for MRI	BI-RADS category	Histology type	Subtype	Size (mm)	Staging
51	Screening – BRCA2 mutation	4	Invasive lobular carcinoma	Luminal B	3.0	T1N0
42	Screening – P53 mutation	4	Invasive ductal carcinoma	Her2+	8.0	T1N0
77	Screening – family history of breast cancer	4	Mucinous carcinoma	Her2+	5.0	T1N0
56	Previous breast cancer + P53	4	Invasive ductal carcinoma	Luminal B	6.0	T1N0
45	Previous breast cancer + P53	4	Invasive ductal carcinoma	Luminal B, Her2+	4.0	T1N0
59	Previous breast cancer	4	Invasive ductal carcinoma	Triple-negative	15.0	T1N0
37	Previous breast cancer	4	Invasive ductal carcinoma	Luminal B	1 7.0	T1N0
32	Previous breast cancer	4	DCIS	—	13.0	Carcinoma *in situ*
39	Screening – previous radiotherapy	5	Invasive ductal carcinoma	Luminal B	30.0	T2N1
75	Screening – no known risk factors	5	Invasive ductal carcinoma	Luminal B	13.0	T1N0
51	Previous breast cancer	5	Invasive ductal carcinoma	Triple-negative	15.0	T1N0

### Follow-up

In 464 (77.7%) of the 597 cases, the patients were followed for at least one year. In five of those cases (Table 6), malignant tumors were identified within the first year after the date of the MRI evaluated (interval cancer). Those included three cases of DCIS identified only by microcalcifications on mammography (one on the same date as the MRI, and the two others at four and six months after the MRI); one case of invasive ductal carcinoma identified on ultrasound at 10 months after the MRI; and one case of invasive ductal carcinoma identified on mammography, ultrasound, and MRI, all performed at 10 months after the initial MRI.

**Table 6:** Characteristics of patients in whom malignant tumors were detected within the first year after the initial breast MRI.

Age (years)	Indication for MRI	Tyrer-Cuzick risk	Diagnostic imaging	Histology type	Subtype	Size (mm)	Staging
40	Screening – BRCA1 mutation	—	MG/US/MRI	Invasive ductal carcinoma	Triple-negative	16.0	T1N0
58	Previous breast cancer	15–20%	US	Invasive ductal carcinoma	Luminal B	16.0	T1N0
37	Screening – BRCA2 mutation	—	MG	DCIS	—	21.0	Carcinoma *in situ*
46	Screening – family history of breast cancer	15–20%	MG	DCIS	—	4.0	Carcinoma *in situ*
43	Screening – CHECK2 mutation	—	MG	DCIS	—	20.0	Carcinoma *in situ*	

## DISCUSSION

### Indications for MRI screening

There is a tendency toward an increase in the number of MRI screening examinations requested in Brazil. In a study carried out by Marques et al.^(14)^, only 8.5% of 529 examinations performed in the 2008–2009 period were for screening. Ferreira et al.^(15)^ evaluated 1,353 breast MRI examinations performed between 2014 and 2018 and reported that 17.4% were indicated for screening. In the present study, 28.0% of the 2,227 examinations carried out in 2020 were indicated for screening. The main indication for MRI screening in our sample was a personal history of breast cancer, followed by a family history of breast cancer and the presence of a known mutation.

Women with a personal history of breast cancer have a risk of recurrence or a second breast cancer of 0.5–1.0% per year within the first 10 years after diagnosis. Although hormone therapy and chemotherapy reduce that risk, women with a history of early-stage estrogen receptorpositive cancer are still at an increased risk of developing cancer^(8)^. The age at diagnosis is important: women who are diagnosed before the age of 50 and undergo breastconserving surgery have been shown to have a ≥ 20% lifetime risk of developing a second breast cancer^(8)^. According to the recommendations of the ACR, it is advisable to consider annual breast MRI screening for women with a personal history of breast cancer, especially for those who have dense breasts or were diagnosed before the age of 50^(8)^. The ACR also recommends that patients at high risk (lifetime risk > 20% of developing breast cancer) should undergo screening, preferably with MRI, at an earlier age.

There are a number of statistical models that are used in order to predict the risk of developing breast cancer^(16)^. In the present study, we used the Tyrer-Cuzick model to assess the risk of breast cancer in 243 patients without a personal history of breast cancer. We found that approximately one third of those patients were at high (> 20%) risk, with patients at low (< 15%) risk accounting for nearly half of the sample. In a large proportion of the cases, no risk factors were identified, only approximately 10% of those cases presenting high risk according to the Tyrer-Cuzick model. These data reflect the need for more widespread use of these risk calculation tools, which could result in more appropriate indication of MRI for breast cancer screening, in Brazil.

In our sample, the main mutations identified were in the P53, BRCA1, and BRCA2 genes, which is in line with the mutations most often observed in the population of Brazil. A study conducted by Guindalini et al.^(17)^ included the largest cohort to date of breast cancer patients undergoing multigene panel testing in Brazil. The authors found that, in that cohort, BRCA1 and BRCA2 mutations accounted for nearly 50% of all germline variants, and the third most common mutation was of the P53 gene. According to the current ACR recommendations, in patients at increased risk for breast cancer based on genetics (those with a BRCA1 or BRCA2 mutation), screening MRI should be performed annually starting at 25–30 years of age.

Other, less common, gene mutations include CHEK2, PALB2, CDH1, MLH1 (associated with Lynch syndrome), and RET. There is less evidence regarding the benefit of MRI screening in patients with those less common mutations than there is regarding its benefit in patients with mutations in the BRCA1 or BRCA2 gene. However, Lowry et al.^(18)^ suggested that annual MRI screening starting at 30–35 years of age, followed by annual MRI and mammography starting at age 40, reduces breast cancer mortality by more than 50% for women with the ATM, CHEK2, or PALB2 pathogenic variant.

Some patients in our study were referred for MRI screening because they had dense breasts. It is known that breast density is an independent risk factor for the development of breast cancer, reducing the sensitivity of mammography for screening. Recent studies report that contrastenhanced breast MRI performed as a screening method in women with extremely dense breasts reduces the mortality rate among such women^(19)^. Currently, both the ACR and the European Society of Breast Imaging recommend MRI screening in patients with dense breasts^(8,19)^.

### MRI screening results

An audit of the medical results is essential to the evaluation of the results of screening tests^(20)^. The rigorous use of the ACR and BI-RADS terminology and recommendations is essential to allow accurate data capture and coding^(13)^. Breast MRI data should be collected and reported in a manner similar to that employed for mammography data. The screening results in our study are in accordance with the reference values established in the BI-RADS and in the literature, confirming that MRI is a highly accurate method for early detection of malignant neoplasms in this population.

Sedora Román et al.^(21)^ conducted a retrospective study with the aim of auditing breast MRIs performed between 2011 and 2013. They found that the abnormal interpretation rate for screening examinations ranged from 8% to 17%. In the present study, the abnormal interpretation rate was 28.8%, higher than that reported by those authors, mainly because a greater number of MRI findings in our sample (24% of the total) were classified as BI-RADS category 3. This high number of MRI findings classified as BI-RADS 3 can be explained by the fact that the facility is a referral center for cancer, where there are high numbers of high-risk patients and patients undergoing oncological follow-up, and that radiologists tend to give greater weight to certain findings among such patients. These data are similar to those published by Niell et al.^(22)^, who reported a high proportion of BI-RADS 3 MRI findings, which is consistent with data in the literature showing that the frequency of BI-RADS 3 classification decreases in relation to increases in the number of serial breast MRI examinations and in the level of experience of the radiologist.

In our sample, the cancer detection rate on MRI was 18.2/1,000 examinations, which is slightly below the BIRADS benchmark of 20–30/1,000 examinations but similar to the 14–24/1,000 examinations reported in other studies in the literature^(20,21,22)^. The PPV3 in our study was 37.9%, within the 20–50% range suggested in the BI-RADS, albeit higher than the 21–27% reported elsewhere^(20,21,22)^.

In our sample, MRI screening identified cancer in 11 patients, who ranged in age from 32 to 75 years. The neoplasms identified in the screening were small and in the early stages. Five patients with malignant tumors were identified within the first year after the date of the MRI evaluated, with three cases of DCIS being identified only by microcalcifications on mammography. Chiarelli et al.^(23)^ demonstrated that not performing mammography in these patients can reduce the rate of detection of DCIS, especially that of low- or intermediate-grade DCIS.

### Limitations and future perspectives

In this study, we analyzed the examinations carried out at a reference center for cancer over a one-year period. However, those examinations were carried out opportunistically, without an organized screening program, which may have had an impact on the results. Because it was a retrospective study, the medical records of some patients were incomplete, there was a lack of data regarding the indication for MRI screening, there was no standardized follow-up of all cases, and some patients were lost to follow-up. Of the patients in our sample, more than half (59.3%) had a history of breast cancer. The fact that the Tyrer-Cuzick model does not contemplate patients with a history of breast cancer hindered the calculation of the risk of developing a new cancer or locoregional recurrence in those patients.

Currently, there is a trend toward the development of screening protocols that are more personalized, with the best screening strategies being based on the characteristics of patients and on the assessment of their individual risk for developing breast cancer. However, we still need better predictive models to more accurately define the risk of developing breast cancer. Tools using genetic tests or even artificial intelligence have produced promising results and could be incorporated into clinical practice. Recent advances in breast MRI, especially the use of abbreviated protocols, could reduce the cost of the examination so that it is available to more women, while maintaining high diagnostic accuracy^(24)^.

## CONCLUSIONS

The main indication for MRI screening in our sample was a personal history of breast cancer, followed by a family history of breast cancer and the presence of a known mutation, with the most frequent mutations in our sample being P53, BRCA1, and BRCA2. Our screening results are in agreement with the values reported in the literature, with a cancer detection rate of 18.4/1,000 examinations, a PPV3 of 37.9%, a proportion of minimal cancers of 54.5%, and a proportion of invasive carcinomas with node-negative axillae of 90.9%. These findings confirm the importance of MRI in screening patients at high risk of breast cancer in Brazil, as a tool for early detection of the disease in asymptomatic women, which can improve the effectiveness of treatment and could reduce mortality rates.
